# The Aflatoxin M1 Content of Cow Milk in Cyprus as Affected by Season and Year of Production: A Five-Year Survey

**DOI:** 10.3390/foods15132347

**Published:** 2026-07-02

**Authors:** Artemis P. Louppis, Michalis S. Constantinou, Michael G. Kontominas

**Affiliations:** Laboratory of Food Chemistry, Department of Chemistry, University of Ioannina, 45100 Ioannina, Greece

**Keywords:** aflatoxin M_1_, cow milk, mycotoxins, milk contamination, seasonal variation, food safety, risk assessment, Cyprus

## Abstract

Aflatoxin M1 (AFM1) is of primary importance to the food industry, state inspection authorities and consumers alike due to the carcinogenic nature of this toxin and the respective health risk associated with its presence in dairy products. In the present work, 1197 raw cow milk samples were collected and analyzed for AFM1 in the Republic of Cyprus during a five-year period (2021–2025) using the enzyme-linked immunosorbent assay (ELISA), confirmed by liquid chromatography-mass spectrometry (UPLC-MS/MS). Health exposure assessment and risk characterization were also performed for the Cypriot population (men, women and children) regarding milk consumption through the calculation of Estimated Daily Intake (EDI), Hazard Index (HI) and Margin of Exposure (MoE). Results showed that AFM1 in cow milk varied with season: 7.34 ± 8.59 ng/kg for winter, 6.86 ± 10.00 ng/kg for spring, 6.43 ± 7.44 ng/kg for summer and 5.36 ± 7.11 ng/kg for autumn. Among the analyzed years, 2022 and 2025 showed wider concentration ranges, with several samples presenting elevated AFM1 levels compared to the other years. Of the 1197 milk samples analyzed for AFM1 content, 633 were <LOD, 284 < LOQ, 278 at concentrations of 10–50 ng/kg, and only two samples exceeded the limit set by the EU (50 ng/kg). The average amount of AFM1 exposure EDI ranged between 0.026 ng/kg b.w./day for men and 0.061 ng/kg b.w./day for children. HI was <1, recording values of 0.130 for men, 0.155 for women and 0.305 for children. Finally, MoE recorded values > 10,000 (15,385 for men and 12,903 for women) and 6557 for children, indicating that the amount of AFM1 consumed through milk by children may comprise a considerable risk for this population group and consequently AFM1 contamination of milk demands its regular monitoring and evaluation of the respective risk involved.

## 1. Introduction

**Figure 1:**
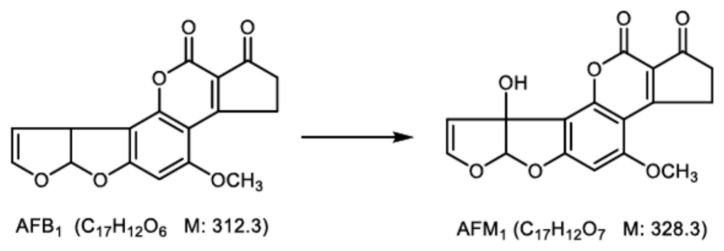
Aflatoxin B1 conversion into Aflatoxin M1 in the animal liver.

Cow milk is a nutritious staple food with an average composition of water (87%), high-quality proteins (3%), lipids (3–4%), lactose (4–5%), minerals (0.8%), and vitamins (0.1%) [1]. In addition to nutrition, milk acts as a carrier of antimicrobial components, immunity enhancers (immunoglobulins) and enzymes [2]. The significance of milk in a balanced diet is obvious, as it comprises the first food for humans, providing a nutrient-complete nourishment during early life. Milk, along with cheese and yogurt, comprises a nutrient-dense food for human nutrition [3]. Mycotoxins are metabolites of filamentous fungi, mainly of the *Aspergillus*, *Penicillium*, and *Fusarium* spp., and more specifically, of *Aspergillus flavus*, *Aspergillus parasiticus*, and, less commonly, by *Aspergillus nomius* [4,5]. According to reports of the Food and Agriculture Organization (FAO), contamination of food with mycotoxins exceeds 25% of the total food supply [6]. Mycotoxins thrive in hot, humid, sub-tropical and tropical climates [7]. Apart from heat and humidity, the growth of aflatoxigenic fungi is essentially influenced by methods and duration of grain storage as well as other agricultural practices [8]. Aspergillus-produced mycotoxins enter both the feed and food chain at numerous pre-harvest or post-harvest points of cereal grain production, including wheat, maize, barley, rice and oats, as well as through nuts, cocoa, dried fruit, peanuts, pistachio, oilseeds and spices [9]. Excessive levels of aflatoxins (AFs) in food comprise a major concern, especially in non-industrialized countries [10]. Types of aflatoxins commonly implicated in food contamination include aflatoxins B1, B2, G1, and G2 [11]. Of these, AFB1, the most toxic and potent aflatoxin, usually contaminates animal feed. The most frequent contamination by AFB1 has been reported in peanuts and corn, with the latter comprising a major component of animal feed [12]. Consumption of feed contaminated with AFB1 by lactating animals converts AFB1 to AFM1 in the animal liver (Figure 1). AFM1 is the main mono-hydroxylated derivative of AFB1 [13]. AFM1 is excreted in both milk and urine [13]. The amount of AFM1 excreted into the milk varies between 1 and 6% of the AFB1 ingested by the animal, depending on cow breed and amount of milk produced [14].

A number of factors affect the amount of carry-over from feed to milk in dairy cows, including ingestion rate, digestion rate, feeding regimes, hepatic biotransformation capacity, animal health, and amount of milk produced [15]. Exposure of humans to AFM1 has been documented to cause carcinogenicity, mutagenicity, hepatotoxicity and teratogenicity [16]. AFM1 also adversely affects the immune system and is responsible for substantial economic losses [12]. For the above reasons, AFM1 exposure through the consumption of milk poses a major concern for humans, particularly for infants and children whose diets are based on milk, and consequently, are more vulnerable to its negative effects. AFB1 has been classified as a Group 1 human carcinogen by the International Agency for Research on Cancer (IARC) [17]. It should be noted that even though legislation regarding the control of AFM1 exists in more than 80 countries, such legislation is not harmonized internationally. For example, the USA (USFDA) and China have adopted an AFM1 maximum residue limit (MRL) in milk of 500 ng/kg [18], whereas the European Union (EU) has adopted a stricter MRL of 50 ng/kg for maximum consumer protection [19]. High contamination percentages of maize with AFs have been reported in several countries of southern and southwestern Europe, including Spain, Italy, Serbia, Croatia [20], as well as Middle Eastern countries, such as Iran, Egypt and Syria [21]. Specifically for Serbia, a major outbreak regarding AFM1 contamination in milk was reported in 2013, in which a large number of milk and dairy samples exceeded the EU MRL limit. The incident was attributed to adverse climatic conditions in 2012, which were responsible for the increased AFB1 contamination of animal feeds used to feed lactating animals. The outbreak resulted in a huge number of product recalls and a dramatic drop in the purchase of dairy products by consumers. More specifically, Popovic et al. [22] reported a total loss of EUR 74.7–96.2 million in the Serbian economy during this two-year crisis. Likewise, 45% of milk samples analyzed in India contained AFM1 exceeding the MRL limit [23]. In another study, 62% of raw milk samples from small farms and 31% from industrial farms in Pakistan were found to be positive for AFM1 [24]. Based on the above, it is of utmost importance to monitor milk supplies for the presence of AFM1 so as to minimize health risks associated with the deleterious effects of this hazardous substance. Considerable research has been carried out on the most suitable methods for the determination of AFM1 in milk and dairy products, concluding that (i) high performance liquid chromatography with fluorescence detector (HPLC-FLD) (adopted as the reference method), (ii) HPLC-MS/MS and (iii) ELISA are three methods that are satisfactory in meeting the analytical and legal requirements for the reliable determination of AFM1 in routine monitoring programs, all showing a strong correlation among them. Of the three, the ELISA method is the simplest and the most rapid for the reliable determination of AFM1 [5,25]. Furthermore, ELISA is a high-throughput assay with low sample volume requirements, along with fewer needs for sample clean-up compared to HPLC methods. Both ELISA and HPLC-FLD comprise ISO standard methods (ISO/IEC 17025:2018) [26].

The objectives of the present study were (1) to evaluate the effect of season and year on the contamination of milk with AFM1 in the Republic of Cyprus over a period of 5 years (2021–2025), (2) to estimate the potential health risk of the Cypriot population associated with the exposure through milk consumption and (3) to use obtained analytical data to justify or not the need for the systematic monitoring of AFM1 in milk and dairy products in Cyprus. To our knowledge, this is the first study on the systematic monitoring of AFM1 in the Republic of Cyprus.

## 2. Materials and Methods

### 2.1. Sample Collection

Raw cow milk samples were obtained from dairy farms (sampling points) located throughout the Republic of Cyprus, including the regions of Nicosia, Larnaca, Limassol, and Pafos, during the period from January 2021 to December 2025 as part of routine monitoring programs by trained personnel using a dedicated sterile sampling vessel. Overall, 1197 milk samples were analyzed throughout the study period, comprising 224 samples collected in 2021, 218 in 2022, 261 in 2023, 216 in 2024, and 278 in 2025. For each sampling point, approximately 50 mL of raw milk was collected in sterile containers and stored frozen at −18 °C. At the end of each season, collected samples were transported by air in ice boxes on ice within 6 h to the laboratory and stored at 4 ± 1 °C until analysis.

### 2.2. Chemicals and Reagents

The determination of aflatoxin M1 (AFM1) was carried out using a commercially available competitive enzyme-linked immunosorbent assay (ELISA) kit (Bio-Shield M1 ES ELISA, Prognosis, Larissa, Greece), following the manufacturer’s protocol and the requirements outlined in EN ISO 14675:2003 [27]. The assay kit contained microplate wells pre-coated with anti-AFM1 antibodies, AFM1 calibration standards at concentrations of 0, 5, 10, 25, 50, 100, and 250 ng/kg, enzyme conjugate reagent, a 20× concentrated wash buffer, tetramethylbenzidine (TMB) substrate solution, and a stop reagent consisting of 15% phosphoric acid.

AFM1-free milk supplied with the ELISA kit was used as the negative control. All kit reagents were maintained at 2–8 °C prior to analysis. Prior to analysis, the concentrated wash buffer was diluted with deionized water to prepare the working washing solution. A certified AFM1 standard solution (10 mg/L) was prepared in acetonitrile using reference material obtained from HPC (Jesewitz, Germany) and stored at −18 °C. Working calibration solutions were subsequently prepared by diluting the stock solution with AFM1-free milk immediately before analysis.

### 2.3. Determination of Aflatoxin M1

Before analysis, milk samples were placed into 50 mL centrifuge tubes and centrifuged at 3000× *g* for 10 min using a Sigma 2–16 KL centrifuge (Osterode am Harz, Germany) to facilitate fat separation. The upper fat layer was then carefully aspirated with a glass pipette, and the resulting defatted milk was subjected to analysis. Subsequently, 100 μL aliquots of calibration standards and prepared samples were dispensed in duplicate into microplate wells pre-coated with AFM1-specific antibodies and incubated for 45 min at room temperature. Following incubation, the microplate wells were washed four times with washing solution to eliminate unbound material. Next, 100 μL of enzyme conjugate reagent was added to each well, followed by incubation at room temperature for 15 min. After an additional washing step, 100 μL of tetramethylbenzidine (TMB) substrate solution was introduced into each well, and the plate was kept in the dark for 15 min to allow for the color to develop. The enzymatic reaction was terminated by adding 100 μL of stop solution containing 15% H_3_PO_4_, producing a yellow coloration in the wells. Absorbance was subsequently recorded at 450 nm using a microplate reader (Biotek 800-TS, BioTek Instruments, Agilent Technologies, Santa Clara, CA, USA). Quantification of AFM1 was achieved through a calibration curve generated from AFM1 standard solutions in the range of 0–250 ng/kg. The calibration data were processed using an exponential regression model incorporated in the ELISA data analysis software supplied by Prognosis (Larissa, Greece). All samples were examined in duplicate, and the obtained results were reported in ng/kg (ppt). The method exhibited a limit of detection (LOD) of 3 ng/kg and a limit of quantification (LOQ) of 10 ng/kg.

The performance of the ELISA screening method was verified under routine laboratory conditions at three AFM1 concentration levels: 10, 20, and 50 ng/kg. Mean recoveries were 113.5%, 113.0%, and 96.8%, respectively. Repeatability, expressed as RSDr, ranged from 6.5% to 11.5%, while within-laboratory reproducibility/intermediate precision ranged from 6.2% to 13.1%. Trueness values ranged from 0.0% to −3.2%, and the expanded uncertainty ranged from 15.6% to 27.8%. These results confirmed the suitability of the ELISA method for routine AFM1 screening in cow milk samples. Quality control was ensured through the analysis of AFM1-free milk as a negative control, fortified milk samples at three concentration levels, and duplicate analysis of all samples.

Samples containing AFM1 concentrations above the regulatory threshold of 50 ng/kg were additionally verified using an in-house developed QuEChERS extraction followed by UPLC-MS/MS analysis. In brief, 10 mL of raw milk was extracted with 10 mL of acetonitrile containing 0.5% formic acid using vortex agitation for 5 min. Phase separation was achieved by adding 8 g MgSO_4_ and 1.2 g NaCl, followed by vortex mixing for 1 min and centrifugation at 3134× *g* for 10 min. The acetonitrile phase was subsequently collected. A 2 mL portion of the extract was subjected to dispersive solid-phase extraction (d-SPE) clean-up using 900 mg MgSO_4_ and 300 mg C18 sorbent. After centrifugation for 5 min at 3134× *g*, the purified extract was evaporated to dryness and reconstituted in 150 µL of the same extraction solvent, resulting in a 10-fold concentration factor. Finally, the solution was passed through a 0.22 µm filter before instrumental analysis. Chromatographic analysis of AFM1 was carried out using an ACQUITY I-Class UPLC system (Waters, Milford, MA, USA) interfaced with a Xevo TQ-Absolute triple quadrupole mass spectrometer (Waters Corporation, Milford, MA, USA) equipped with an electrospray ionization (ESI) source. Separation was performed on a Waters ACQUITY HSS T3 analytical column under gradient elution conditions. The mobile phase consisted of (A) water containing 0.1% formic acid and 5 mM ammonium formate and (B) methanol. The elution program was initiated at 0% B, increased to 14% within 0.5 min and maintained for 1.5 min, and followed by an increase to 60% B over 1 min with a subsequent hold of 0.5 min. The proportion of solvent B was then gradually raised to 100% over 4.5 min and maintained for 2 min before re-equilibration to the starting conditions. The flow rate was set at 0.3 mL/min, the column oven temperature at 40 °C, and the injection volume at 5 μL. Detection of AFM1 was performed in positive ionization mode using a precursor ion of *m*/*z* 329 and product ions of *m*/*z* 273 and 259, with the compound eluting at approximately 6.0 min.

The UPLC-MS/MS confirmatory method was verified under routine laboratory conditions at three AFM1 concentration levels: 10, 20, and 50 ng/kg. Mean recoveries were 108%, 97.3%, and 102.1%, respectively. The calibration curve was linear over the range of 250 ng/kg, with a correlation coefficient R^2^ = 0.99987. Matrix effect was evaluated by comparing the slopes of matrix-matched and solvent-based calibration curves and was found to be 15%. The LOD and LOQ of the UPLC-MS/MS method were 0.3 ng/kg and 1.0 ng/kg, respectively. Repeatability, expressed as RSDr, ranged from 3.5% to 9.4%, while within-laboratory reproducibility/intermediate precision ranged from 4.2% to 9.1%. Trueness values ranged from 0.0% to 2.1%, and the expanded uncertainty ranged from 9.1% to 15.6%. These results confirmed the suitability of the UPLC-MS/MS method for confirmatory determination of AFM1 in cow milk samples.

### 2.4. Risk Assessment

The risk assessment of AFM1 in a given foodstuff (milk) involves calculating the Estimated Daily Intake (*EDI*) based on mean contamination levels and consumption rates, followed by risk characterization using the Margin of Exposure (*MoE*) or Hazard Index (*HI*) approach. Because AFM1 is a genotoxic carcinogen, the MoE approach is favored by the European Food Safety Authority (EFSA).

#### 2.4.1. Calculation of Estimated Daily Intake (EDI)

The risk of exposure to AFM1 through milk consumption was calculated using Equation (1):

(1) $$\mathrm{EDI}\;(\mathrm{ng}/\mathrm{kg}\;b.w./\mathrm{day})\;=\;\frac{DMI\;\times \;C}{b.w.}$$

where *DMI* is the daily milk consumption (L/day), *C* (ng/kg) is the mean AFM1 concentration in milk samples (ng/kg), and b.w. is the average body weight of consumers (70 kg for men, 60 kg for women and 30 kg for 12-year-old children).

#### 2.4.2. Risk Characterization Approaches

##### Hazard Index (HI) Approach for Non-Carcinogenic Risk

Based on an average body weight, the *HI* of AFM1 exposure through milk intake was calculated based on the *EDI* and tolerable daily intake (*TDI*) for AFM1 using Equation (2):

(2) $$HI=\frac{EDI\;(\frac{ng}{kg}b.w./day)}{TDI\;(\frac{ng}{kgb}b.w./day)}$$

The *TDI* for AFM1 was considered 0.2 ng/kg body weight per day based on the previous studies. This value was derived in older and recent literature [28,29] by dividing the median toxic dose (*TD*_50_) by an uncertainty factor of 5000.

*HI* values > 1 indicate a significant health risk of exposure to AFM1 in the milk-consuming population [28].

#### 2.4.3. Margin of Exposure (MoE) Approach for Carcinogenic Risk

For genotoxic substances, no tolerable daily intake is set. The *MoE* compares the toxicological reference point (*BMDL*_10_) to the estimated exposure through Equation (3):

(3) $$\mathrm{MoE}=\;\frac{BMLD10}{EDI}$$

where *BMDL*_10_ is the critical effect dose of a toxic substance that increases the incidence of liver cancer (*HCC* = hepatocellular carcinoma) by 10%.

Values of *MoE* < 10,000 reflect an alarming risk level of *HCC* induced by AFM1 exposure. In the *MoE* approach and based on studies in animals, a *BMDL*_10_ of 0.4 μg/kg b.w. per day for the incidence of *HCC* in male rats following AFB1 exposure is to be used. Since the *BMDL*_10_ level is not determined for AFM1, EFSA approved the use of a potency factor of 0.1 in combination with the *BMDL*_10_ of 0.4 μg/kg b.w. per day for the induction of *HCC* by AFB1, for the AFM1 risk assessment [29]. Thus, in the present study, a *BMDL*_10_ value of 4 μg/kg b.w. per day was used for the AFM1 risk assessment.

### 2.5. Statistical Analysis

Descriptive statistical analysis was applied to assess the occurrence and distribution of AFM1 in the analyzed milk samples. Average AFM1 concentrations and corresponding standard deviations were determined according to both production season and year. Mean AFM1 concentrations and corresponding standard deviations were determined according to both production season and year. To investigate seasonal fluctuations in AFM1 contamination, the collected samples were categorized into four seasonal groups: winter, spring, summer, and autumn. Variations in AFM1 concentrations between seasons were statistically examined using the non-parametric Kruskal–Wallis test. Furthermore, year-to-year variability was assessed by comparing AFM1 concentrations across the different sampling years (2021–2025). Differences were considered statistically significant at a *p*-value lower than 0.05. When the Kruskal–Wallis test indicated statistically significant differences, Dunn’s post hoc multiple comparison test with Bonferroni correction was performed to identify significant pairwise differences between seasons and years. For statistical analysis and exposure assessment, left-censored AFM1 results were treated using a substitution approach. Samples with concentrations below the LOD were assigned a value of LOD/2, while samples with concentrations between the LOD and LOQ were assigned a value of LOQ/2. Quantifiable samples were included using their measured AFM1 concentrations. This approach was applied for the calculation of mean concentrations, standard deviations, Kruskal–Wallis statistical comparisons, and the risk assessment indices EDI, HI, and MoE. In parallel, occurrence data were reported separately according to the analytical categories <LOD, <LOQ, 10–50 ng/kg, and >50 ng/kg. No missing AFM1 concentration values were present in the final dataset used for statistical analysis.

## 3. Results and Discussion

### 3.1. Occurrence of AFM1 in Milk Samples

A total of 1197 raw cow milk samples were collected across Cyprus between 2021 and 2025 and used for the determination of AFM1 (Table 1). The number of samples analyzed each year ranged from 216 to 278, ensuring a representative dataset covering multiple production years and seasonal periods. Results on the amounts of AFM1 recorded in milk samples are summarized in Table 2.

**Figure 2:**
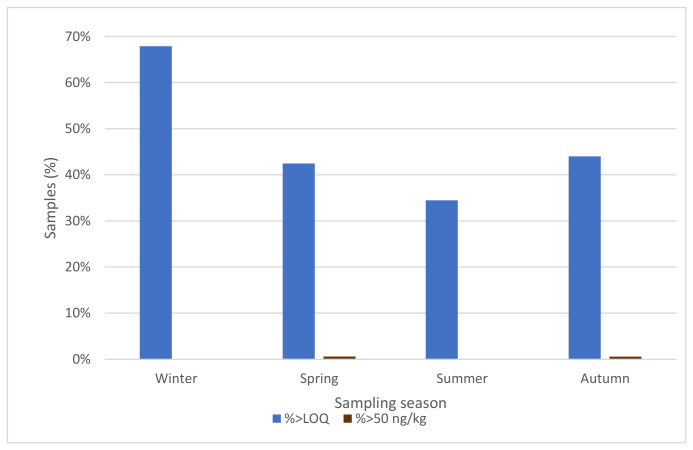
Occurrence of AFM1 above LOQ and EU limit (50 ng/kg) in Cypriot raw cow milk samples by season.

The majority of samples (633 samples; 52.9%) contained AFM1 concentrations below the method limit of detection (LOD = 3 ng/kg), indicating that more than 50% of the milk samples analyzed showed no detectable contamination. In addition, 284 samples (23.7%) presented concentrations between the LOD and LOQ (10 ng/kg), suggesting the presence of trace amounts of AFM1 that could not be quantified with sufficient analytical precision. AFM1 concentrations within the quantifiable range of 10–50 ng/kg were detected in 278 samples (23.2%), indicating that approximately one quarter of the analyzed milk samples contained measurable levels of AFM1. Despite the relatively frequent measurement of low concentrations, only two samples (0.17%) exceeded the EU MRL established by the EU for milk intended for human consumption. The distribution of samples exceeding the LOQ and the EU regulatory limit is illustrated in Figure 2, confirming that although AFM1 was determined in a proportion of the analyzed samples, exceedances of the legal limit were extremely rare. This contamination is typically associated with the presence of AFB1 in animal feed, which can originate from fungal growth (i) in contaminated cereals, (ii) compound feed, or (iii) stored feed materials under inappropriate conditions. Following ingestion by dairy cattle, AFB1 is metabolized in the cow liver and partially converted into AFM1, which is eventually excreted in milk [13]. Consequently, the presence of AFM1 in raw or pasteurized milk is indicative of the degree of contamination of the feed used in dairy production in Cyprus [30]. The very low percentage of samples above the regulatory threshold indicates a high level of compliance with European food safety standards for milk in Cyprus. The results of the present study, with regard to the level of milk contamination by AFM1, are in partial agreement with those of the recent literature considering the country they originate from:

Sadighara et al. [12] collected milk samples from 24 farms in Iran in the winter of 2021 and determined contamination with AFM1 using the HPLC-FLD method. The authors reported AFM1 amounts in the range of non-detectable to 160 ng/kg. Most of the samples recorded AFM1 values lower than the national and EU MRL limit. In 16% of the samples, the concentration of AFM1 exceeded the MRL limit. Ferrari et al. [5] collected 95,882 samples of whole raw milk from northern Italy between 2013 and 2021 and analyzed them for AFM1 using the ELISA method. Results showed that only 667 out of 95,882 samples analyzed (0.7%) showed AFM1 concentrations higher than the EU MRL limit; 390 samples (0.4% of total samples) showed AFM1 values between 40 and 50 ng/kg, thus requiring corrective action despite not exceeding the EU MRL limit. Lin et al. [31] collected 44 samples of fresh milk, 45 samples of milk powder and 24 samples of drinking yogurt from various outlets in Taiwan from June to August 2002, and determined product contamination with AFM1. AFM1 was detected in 40 samples of fresh milk at a concentration range of 0.002–0.083 ng/kg. AFM1 was not found in any of the milk powders. AFM1 was determined in three samples of drinking yogurt, at the levels of 0.007, 0.009 and 0.044 ng/kg. All 113 samples collected in this study met national and EU regulation requirements. Topi et al. [10] determined AFM1 contamination of 119 cow milk samples collected from retail stores in Albania during the years 2019 and 2020. Upon pasteurization or ultra-high temperature (UHT) treatment, samples were analyzed for AFM1 content using the ELISA method. Results showed that milk contamination with AFM1 was higher in pasteurized milk compared to UHT-treated milk (59.68% and 43.86%, respectively). AFM1 was not detected in 57 out of 119 milk samples (47.89%) (<LOD = 5 ng/kg). The AFM1 concentrations ranged between 5 and 10 ng/kg in 34 milk samples (28.57%); between 11 and 49 ng/kg in 21 milk samples (17.65%), and exceeded the EU MRL limit in 7 milk samples (5.88%). Regarding the effect of year, milk samples from 2019 showed higher AFM1 contamination (81.36%) than those from 2020 (23.33%) attributed to specific climatic conditions during that year. Maggira et al. [32] collected 396 milk samples originating from cow, goat, and sheep directly from producers across Greece and determined AFM1 contamination using the ELISA and, selectively, the HPLC-FLD method in an effort to compare the two. Results of the ELISA method showed that AFM1 was determined in 39 samples (10.15%) with concentrations below the EU MRL limit, with only three samples (0.75%) exceeding this limit. Between the two methods, ELISA proved to be faster and equally reliable compared to HPLC for the determination of AFM1 in milk. Roila et al. [33] collected 16,934 cow and ewe’s milk samples in central Italy from 2014 to 2020 and analyzed them for the presence of AFM1 by the ELISA method, also confirmed by HPLC-FLD. AFM1 concentration ranged from 9 to 15 ng/kg in cow milk. The respective concentration in ewe milk ranged from 9 to 13 ng/kg, all meeting the EU regulation requirement. Serraino et al. [14] collected 31,702 milk samples from six dairy plants located in Northern, Central, and Southern Italy from April 2013 to December 2018. Sample analysis for AFM1 was carried out using the ELISA method. The monthly average AFM1 contamination of milk samples ranged between 7.19 and 22.53 ng/kg. The results of this study showed a low risk of *HCC*, yet the inter-year variability of climatic conditions, which influence AFB1 contamination of feed and therefore AFM1 contamination of milk, justifies the routine monitoring of milk and continuous update of the risk assessment. Finally, Guo et al. [34] investigated water buffalo milk contamination with AFM1. A total of 136 raw milk samples were collected from dairy pastures and 86 dairy products from supermarkets in south China during October 2016 and March 2017. An analysis of AFM1 was carried out using ELISA and verified using HPLC-FLD. AFM1 was detected in 85 (62.5%) milk samples with concentrations ranging between 4 and 243 ng/kg. Eight samples (5.9%) exceeded the EU MRL limit. AFM1 was detected in 64 (74.4%) of the dairy product samples with concentrations ranging between 4 and 235 ng/kg. AFM1 content (43.1 ng/kg) in cheese was significantly higher than that in pasteurized milk, UHT milk and yogurt. All the milk and dairy products’ AFM1 content was below the Chinese national standard (500 ng/kg). This study concluded that AFM1 contamination in buffalo milk and dairy products does not raise a significant public health issue in South China.

Based on the above, the incidence of AFM1 contamination is considerably lower in developed countries compared to developing countries due to the existence of specific legislation, official analytical methods and monitoring programs. In different countries of North, Central and South America, AFM1 contamination in milk was found to be as high as 64.3%, with 11.2% of the samples containing AFM1 at concentrations exceeding the EU MRL limit, with the highest AFM1 concentration of 7.7 μg/kg recorded in raw milk from Mexico [35]. Likewise, out of 5577 milk samples originating from Asian countries, AFM1 contamination was detected in 4227 (76% of samples), while 28.2% of samples contained AFM1 at concentrations exceeding the EU MRL limit. Of 3140 milk samples tested in Iran, 2806 (89.3%) were found to be positive for AFM1, while 31% of the samples exceeded the EU MRL limit. Compared to Iran, the contamination of milk with AFM1 in China was lower. Finally, the AFM1 content in milk samples was found to range from 0.025 to 1.101 μg/kg in Turkey; 0.020 to 3.090 μg/kg in Pakistan and 0.01 to 0.03 μg/kg in Brazil [36].

Overall, comparison with the available literature indicates that AFM1 contamination in raw cow milk from Cyprus was generally lower than that reported in several countries where higher exceedance rates have been associated with warmer climates, inadequate feed storage, or less systematic monitoring. In contrast, the low percentage of non-compliant samples observed in the present study is closer to findings from countries with established regulatory surveillance and routine control of milk and feed safety. Therefore, the present results suggest that, although AFM1 contamination may occur sporadically, the overall contamination profile of Cypriot raw cow milk is consistent with a relatively well-controlled dairy production chain.

### 3.2. UPLC-MS/MS Confirmation of Non-Compliant Samples

ELISA was applied as a rapid and cost-effective screening method for the determination of AFM1 in cow milk samples. In particular, samples exceeding 50 ng/kg (EU maximum limit) exhibited excellent concordance, with a mean deviation of ±3% and no false negatives detected, supporting the reliability of ELISA for detection of AFM1 in cow milk samples. Since only two samples exceeded the regulatory threshold, the available paired ELISA and UPLC-MS/MS data were limited and were not intended to provide a full method comparison across a wide concentration range. Therefore, ELISA was used as a rapid screening method, while UPLC-MS/MS served as a confirmatory method for samples above the regulatory limit.

### 3.3. Variation of AFM1 According to Season

**Figure 3:**
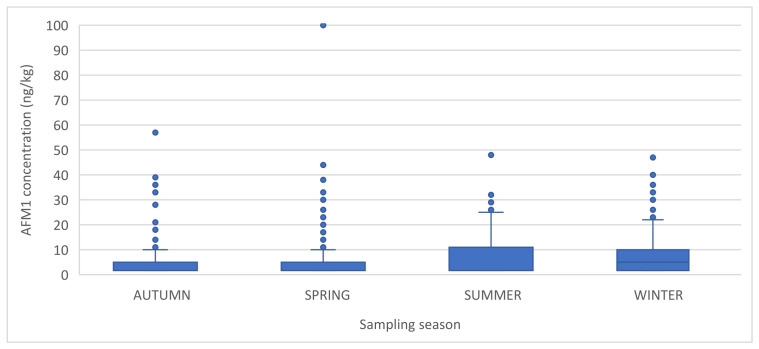
Distribution of AFM1 concentrations in Cypriot cow milk samples according to season.

**Figure 4:**
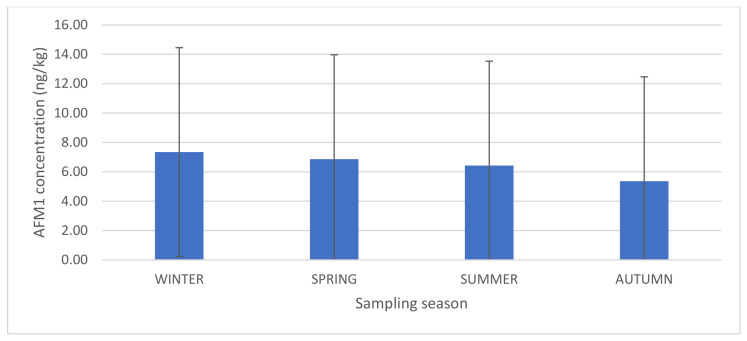
Mean AFM1 concentration (±SD) in Cypriot cow milk samples according to season.

Seasonal variation in AFM1 contamination levels was evaluated using the dataset summarized in Table 2, while graphical representation of the distribution and mean concentrations is presented in Figure 3 and Figure 4, respectively.

Table 2 shows that AFM1 concentrations above the LOQ were recorded in milk samples throughout the year, indicating that AFM1 contamination occurs across all seasons. However, the frequency of quantifiable AFM1 concentrations varied among seasons. The highest absolute number of samples with AFM1 concentrations above the LOQ was observed during summer (77 samples) and spring (74 samples), followed by winter (74 samples) and autumn (55 samples). Despite these differences in occurrence, exceedances of the EU maximum limit of 50 ng/kg were extremely rare, with only two samples exceeding the EU regulatory threshold across all seasons. Seasonal differences in AFM1 concentrations are further illustrated in Figure 3, which presents the distribution of AFM1 concentrations in milk samples according to season. Although AFM1 was detected in samples collected during all seasons, the distribution patterns indicate moderate variability in contamination levels throughout the year. Descriptive statistics presented in Figure 4 show that the mean AFM1 concentrations ranged from 5.36 ng/kg in autumn to 7.34 ng/kg in winter, with intermediate values recorded in spring (6.86 ng/kg) and summer (6.43 ng/kg). While the absolute differences in mean concentrations were relatively small, the variability among individual samples was considerable, particularly in spring (SD = 10.00 ng/kg) and winter (SD = 8.59 ng/kg), suggesting heterogeneity in contamination levels among dairy farms or feed sources. Statistical analysis using the Kruskal–Wallis test demonstrated that AFM1 concentrations differed significantly among seasons (*p* = 0.0031 < 0.05), confirming that seasonal factors influence the occurrence of AFM1 in milk. The seasonal occurrence of AFM1 concentrations above the LOQ and above the EU regulatory limit is illustrated in Figure 2. The figure highlights the proportion of milk samples containing quantifiable AFM1 concentrations during each season. Overall, the highest frequency of samples recording AFM1 concentrations above the LOQ was observed during winter, followed by summer and spring, whereas autumn presented the lowest proportion of quantifiable AFM1 concentrations. This pattern indicates that the likelihood of detecting measurable AFM1 levels in milk may vary throughout the year. Post hoc Dunn’s multiple comparison test with Bonferroni correction showed that AFM1 concentrations differed significantly between winter and spring (*p* < 0.05), and between winter and autumn (*p* < 0.05), whereas the remaining seasonal comparisons were not statistically significant after correction for multiple testing. The observed seasonal variability may be related to differences in feeding practices and climatic conditions affecting the contamination of animal feed with aflatoxin B1. In many dairy production systems, the proportion of stored feed materials such as silage and compound feed increases during colder months, potentially increasing the risk of aflatoxin contamination due to higher humidity levels in autumn and winter, which enhance toxin production, in addition to the fact that the animals are housed during the colder months, consuming mixed feeds rather than fresh forage [37]. Conversely, during periods when fresh forage is more widely available, the likelihood of aflatoxin exposure may be reduced. Despite the statistically significant seasonal variation, the overall AFM1 concentrations remained well below the European Union regulatory limit for milk, indicating that milk produced in Cyprus during all seasons generally complied with EU food safety standards.

Tuncay & Öniz [38] investigated the frequency of AFM1 contamination in raw milk produced in Northern Cyprus during the period of 2018–2020. AFM1 was analyzed using HPLC with fluorescence detection (HPLC-FLD). A total of 1026 raw milk samples were analyzed. Mean contamination with AFM1 was 26.4 ± 17.96 ng/kg. Only 11.4% of the raw milk samples exceeded the EU MRL limit. Milk samples produced in autumn and winter had a much higher contamination incidence (average 31.77 ± 19.21 ng/kg and 26.96 ± 20.77 ng/kg, respectively) in comparison to spring and summer (average 19.00 ± 18.53 ng/kg and 7.51 ± 11.31 ng/kg, respectively). Based on these findings, the authors concluded that it is of utmost importance to continuously monitor the presence of AFM1 in milk, especially during the autumn and winter seasons.

Barzoki et al. [39] collected and analyzed 180 cow milk samples for AFM1 from retail stores in Gorgan, Iran. Each of the 45 samples was collected in October 2022, December 2022, March 2023 and June 2023. The AFM1 content in the samples was determined using the ELISA method. In 139 (72.2%) cow milk samples, the AFM1 content was between 3.5 and 357 ng/kg. All samples recorded Aflatoxin M1 concentrations below the FDA MRL limit of 500 ng/kg. Of these, 41 samples (22.7%) exceeded the EU MRL limit of 50 ng/kg. AFM1 concentrations varied with season: 3.5–237 ng/kg for autumn; 5.4–357 ng/kg for winter; 4.1–243 ng/kg for spring and 4.5–241 ng/kg for summer samples, recording statistically significant differences across different seasons. Considerably lower average AFM1 concentrations were recorded during summer and autumn compared to the winter and spring seasons. The authors attributed such differences to the quantity of mixed feed consumed by cattle during different seasons. The higher concentration of AFM1 in winter was associated with the fact that during this period animals are fed stored feed and feed concentrate rather than fresh forage.

However, this interpretation should be considered with caution, as no feed samples or farm-level environmental data, such as feed storage conditions, temperature, humidity, or feeding regimes, were collected in the present study. Therefore, feeding practices and climatic conditions can only be considered as possible contributing factors rather than confirmed causes of the observed seasonal variation.

### 3.4. Variation of AFM1 According to Year

**Figure 5:**
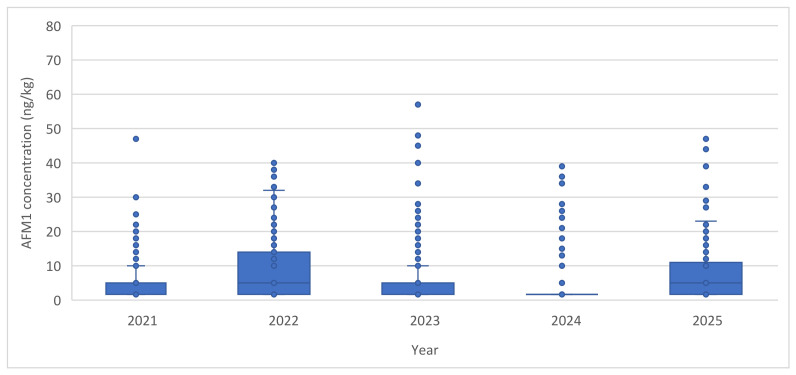
AFM1 concentration in Cypriot cow milk samples according to year (2021–2025).

Interannual variation in AFM1 contamination levels evaluated for the five-year period of 2021–2025 is presented in Figure 5 and Table 1.

Overall, the majority of samples in all years exhibited relatively low AFM1 concentrations, with median values remaining well below 10 ng/kg, confirming that most milk samples contained only low levels of contamination. However, the spread of the distributions varied significantly among years, suggesting differences in the occurrence of higher AFM1 concentrations. Among the analyzed years, 2022 and 2025 showed wider concentration ranges, with several samples presenting elevated AFM1 levels compared to the other years. In contrast, 2024 displayed the lowest variability, with most samples clustered at low concentrations close to the detection limit. The year 2023 exhibited the highest individual concentration values, including samples approaching the regulatory limit. Statistical analysis using the Kruskal–Wallis test revealed a highly significant difference in AFM1 concentrations between the analyzed years (*p* = 2.51 × 10^−13^ < 0.05), indicating that interannual factors influenced the occurrence of AFM1 contamination in milk. Post hoc Dunn’s multiple comparison test with Bonferroni correction identified significant differences between 2021 and 2022 (*p* < 0.05), 2021 and 2024 (*p* < 0.05), 2022 and 2023 (*p* < 0.05), 2022 and 2024 (*p* < 0.05), 2023 and 2024 (*p* < 0.05), and 2024 and 2025 (*p* < 0.05). The remaining pairwise comparisons were not statistically significant after correction for multiple testing. The observed year-to-year variability may be attributed to fluctuations in climatic conditions that influence fungal growth and aflatoxin production in animal feed. Environmental factors such as temperature, humidity, and drought conditions can significantly affect the development of toxigenic *Aspergillus* spp. in cereals and other feed ingredients. Consequently, variations in feed contamination with aflatoxin B1 may lead to differences in AFM1 levels detected in milk among years. Despite the statistically significant interannual variation observed, the majority of AFM1 concentrations remained well below the EU MRL limit of 50 ng/kg, indicating that milk produced in Cyprus during the study period investigated generally complied with regulatory safety standards.

Topi et al. [10] determined cow milk contamination with AFM1 in cows in Albania for 2019 and 2020 and attributed differences in concentrations of AFM1 between the two years to differences in weather conditions prevailing in the country during that period. Ferrari et al. [5] monitored AFM1 contamination in cow milk in northern Italy for an 8-year period, between 2013 and 2021. The highest concentrations of AFM1 were recorded for the years 2013 and 2015, when the average contamination reached 14.4 and 15.1 ng/kg, respectively. Such an increase was consistent with a “peak” in AFB1 contamination in maize in the form of flour and grain during those periods. Serraino et al. [14] determined AFM1 contamination in cow milk in Italy for a five-and-a-half-year period, from 2013 to 2018 and reported similar levels of contamination for years 2014, the first half of 2015, 2017 and 2018. For years 2013, the second half of 2015, and 2016, contamination was highly consistent with an aflatoxin crisis recorded during those periods. Finally, Roila et al. [33] monitored AFM1 contamination in cow milk in central Italy during a 7-year period, ranging between 2014 and 2020, and reported a decreasing trend in the level of AFM1 contamination after 2016, which was considered a year of crisis.

Nevertheless, the interpretation of interannual differences remains limited by the absence of supporting feed and environmental data. Therefore, although climatic variability and feed contamination may have contributed to the observed year-to-year differences, their direct influence could not be confirmed in the present study.

### 3.5. Risk Assessment

Risk assessment was carried out through the determination of indices: *EDI*, *HI* and *MoE*. *EDI*, *HI* and *MoE* were calculated using Equations (4), (5) and (6), respectively. The mean AFM1 concentration used for the calculation of EDI was derived from the same dataset after substitution of left-censored values, as described in the Statistical Analysis section. The following sample calculations are based on winter values corresponding to the worst-case scenario. Per capita consumption of dairy products in Cyprus is high, with an average person consuming 91.6 liters of pasteurized milk (91.6 L) annually, or 0.25 L/day [40].

(4) *EDI_winter_* = 0.25 L/day × 7.34 ng/kg b.w./70 kg = 0.026 for men A total of 0.031 ng/kg b.w. ×/day for women and 0.061 ng/kg b.w. ×/day for children.

Ref. [29]

(5) *HI =* 0.026/0.2 = 0.130 for men 0.155 for women and 0.305 for children

(6) *MoE =* 400 ng/kg b.w. kg/EDI = 15,385 for men, 12,903 for women and 6557 for children.

Results for risk assessment are given in Table 3.

Additional percentile-based exposure scenarios.

As shown in Table 3 and Table 4, the *HI* values corresponding to milk consumption were <1 for adults and children, even though 2 of the 1197 samples (0.17%) had an AFM1 content above the EU MRL limit. With regard to *MoE* values, these were >10,000 for adults but not for children, with recorded values < 10,000. To provide a more conservative characterization of potential consumer exposure, additional percentile-based scenarios were also calculated using the 95th and 97.5th percentile AFM1 concentrations of the dataset. The P95 and P97.5 AFM1 concentrations were 22 and 30 ng/kg, respectively. Under the P95 scenario, EDI values were 0.079, 0.092, and 0.183 ng/kg b.w./day for men, women, and children, respectively. The corresponding HI values were 0.393, 0.458, and 0.917, while MoE values were 5091, 4364, and 2182, respectively. Under the P97.5 scenario, EDI values increased to 0.107, 0.125, and 0.250 ng/kg b.w./day for men, women, and children, respectively. The corresponding HI values were 0.536, 0.625, and 1.250, while MoE values were 3733, 3200, and 1600, respectively. Present results are in line with those of Ghaffarian-Bahraman et al. [29], recording respective *HI* values of 0.270 (for men), 0.307 (for women) and 0.595 (for children) and *MoE* values of 14,833 (for men), 13,044 (for women) and 6719 (for children) regarding AFM1 contamination of milk in southern Iran. With regard to *MoE* values, results indicate that children, due to their larger milk intake and lower body weight compared to adults, comprise the most exposed population group to AFM1, associated with a considerable health risk. This was also shown by Serraino et al. [14], who reported *HI* values of 1.64 and 1.4 for infants and toddlers, respectively, and *HI* values of <1 for adults and adolescents. In this particular study, carried out in Italy, the *EDI* for AFM1 in different population groups ranged between 0.025 and 0.328 ng/kg b.w. per day, in the same order of magnitude as that in the present study. In contrast, Sadighara et al. [12] reported an *HI* index of <1 for both adults and children for AFM1 contamination of milk in Tehran, Iran, concluding that there is no non-carcinogenic risk with respect to the consumption of milk regarding AFM1. In a study by Topi et al. [10], carried out in Albania, *ADD* (average daily exposure) values calculated were 0.082 and 0.096 ng kg/kg b.w./day, higher than those reported in the present study but in the same order of magnitude. Finally, in a study by Roila et al. [33] on milk contamination by AFM1 in central Italy, *MoE* values reported were for the 50th and 99th percentiles of consumption, respectively: 8498 and 2163 for toddlers, 27,301 and 9294 for children, 58,083 and 23,739 for adolescents, 85,277 and 32,437 for adults, and 87,572 and 34,900 for the elderly. The above results on *MoE* values are higher, but in general, agreement with those of the present study indicates that the amount of AFM1 consumed with milk by toddlers and children daily may comprise a considerable risk for these population groups.

## 4. Conclusions

This is the first time that data on the contamination of milk by AFM1 are presented for the Republic of Cyprus during the past five years (2021–2025). A total of 52.9% of 1197 milk samples analyzed showed no detectable contamination (C_AFM1_ < 3 ng/kg); 23.7% of samples recorded concentrations between the LOD and LOQ; 23.2% of samples recorded AFM1 concentrations between 10 and 50 ng/kg, and only 0.17% of samples recorded AFM1 concentrations higher than the EU MRL limit of 50 ng/kg. Risk assessment of AFM1 dietary exposure from cow milk in the population of Cyprus based on the estimation of *EDI*, *HI* and *MoE* indices indicates that children represent the population group requiring the greatest attention in future monitoring, who consume 2× to 3× larger amounts of milk daily compared to adults (average consumption = 0.25 L/day). Taking into consideration (i) the variety of factors affecting the contamination of foodstuffs by aflatoxins and (ii) variability of climatic conditions the planet is experiencing, there is a strong need for the implementation of strict legislation measures and continuous monitoring protocols at all stages of the animal feeding to milk production chain, in order to ensure adequate safety for consumers with regard to the occurrence of AFM1 in their milk supply. Finally, it should be noted that the results of the present study, focusing only on raw milk, do not consider AFM1 intake through the consumption of other dairy products such as cheese and yoghurt. Raw milk represents the primary point of entry of AFM1 into the dairy production chain and is therefore a relevant matrix for routine monitoring and regulatory control. However, processed milk and dairy products may also contribute to total dietary exposure. Such products, due to the concentration step of milk occurring, especially in cheese production, may add notable amounts of AFM1 to the estimated quantity consumed daily. Therefore, future studies including processed drinking milk and dairy products would provide a more comprehensive assessment of AFM1 exposure among consumers in Cyprus.

## Figures and Tables

**Table 1:** Number of Cypriot raw cow milk samples analyzed per year and season (2021–2025).

Year/Season	Number of Samples
Total 2021	224
Autumn	57
Spring	55
Summer	55
Winter	57
Total 2022	218
Autumn	44
Spring	55
Summer	62
Winter	57
Total 2023	261
Autumn	68
Spring	59
Summer	65
Winter	69
Total 2024	216
Autumn	65
Spring	66
Summer	65
Winter	20
Total 2025	278
Autumn	73
Spring	70
Summer	68
Winter	67
Grand Total	1197

**Table 2:** Occurrence of AFM1 in Cypriot raw cow milk samples according to concentration range.

	Number of Samples				
Season	<LOD	<LOQ	>50 ng/kg	10–50 ng/kg	Total Samples
Autumn	175	77	1	54	307
Spring	172	59	1	73	305
Summer	177	61		77	315
Winter	109	87		74	270
Total Samples	633	284	2	278	1197

**Table 3:** Median estimated daily intake (EDI), hazard index (HI), and margin of exposure (MoE) for aflatoxin M1 in males, females, and children.

Index	Male Median	Female Median	Child Median
*EDI* (ng/kg b.w./day)	0.026	0.031	0.061
*HI*	0.130	0.155	0.305
*MoE*	15,385	12,903	6557

*EDI* = Estimated Daily Intake, *HI* = Hazard Index, *MoE* = Margin of Exposure.

**Table 4:** Estimated daily intake (EDI), hazard index (HI), and margin of exposure (MoE) for aflatoxin M1 under high-exposure scenarios.

Scenario	Group	EDI	HI	MoE
P95, 22 ng/kg	Men	0.079	0.393	5091
P95, 22 ng/kg	Women	0.092	0.458	4364
P95, 22 ng/kg	Children	0.183	0.917	2182
P97.5, 30 ng/kg	Men	0.107	0.536	3733
P97.5, 30 ng/kg	Women	0.125	0.625	3200
P97.5, 30 ng/kg	Children	0.250	1.250	1600

*EDI* = Estimated Daily Intake, *HI* = Hazard Index, *MoE* = Margin of Exposure.

## Data Availability

The datasets generated for this study are available on request to the corresponding author.
